# *PCSK9* V474I germline variant drives breast cancer metastasis

**DOI:** 10.1093/lifemeta/loae041

**Published:** 2025-01-04

**Authors:** Hai Wang, Zhiming Shao

**Affiliations:** Key Laboratory of Breast Cancer in Shanghai, Department of Breast Surgery, Fudan University Shanghai Cancer Center; Department of Oncology, Shanghai Medical College, Fudan University, Shanghai 200032, China; Key Laboratory of Breast Cancer in Shanghai, Department of Breast Surgery, Fudan University Shanghai Cancer Center; Department of Oncology, Shanghai Medical College, Fudan University, Shanghai 200032, China

**Breast cancer metastasis remains the leading cause of mortality in patients, yet its mechanisms are poorly understood. A recent *Cell* paper highlights the pivotal role of the proprotein convertase subtilisin/kexin type 9 (*PCSK9*) V474I germline variant in driving metastatic progression through suppression of the low-density lipoprotein receptor-related protein 1 (LRP1) receptor, revealing novel therapeutic opportunities and genetic insights.**

Breast cancer remains one of the most prevalent malignancies worldwide, affecting millions of individuals each year. While advancements in early detection and targeted therapies have significantly improved survival rates for localized disease, metastatic breast cancer remains the leading cause of mortality among patients [1]. Approximately 90% of all cancer-related deaths result from metastasis, emphasizing the critical need to understand and mitigate this complex process. Metastasis is a multi-step cascade involving local invasion, intravasation into blood or lymphatic vessels, survival in the circulation, extravasation at distant sites, and colonization of secondary organs [2]. Despite significant efforts, the precise molecular and cellular mechanisms governing these processes are not fully understood.

Unlike primary tumors, metastases are notoriously resistant to conventional therapies, including chemotherapy, radiotherapy, and even many targeted treatments. This resistance often stems from the biological and genetic heterogeneity of metastatic lesions compared to their primary counterparts [3]. Extensive genomic sequencing of metastatic tumors has failed to identify consistent somatic driver mutations that specifically promote metastasis, suggesting that alternative mechanisms may be at play. In this regard, host factors—genetic variations present in the patient—have emerged as potential contributors to metastatic susceptibility [4]. These factors may exert their influence by modulating the tumor microenvironment, immune response, or interactions between tumor cells and distant organ sites.

**Figure 1:**
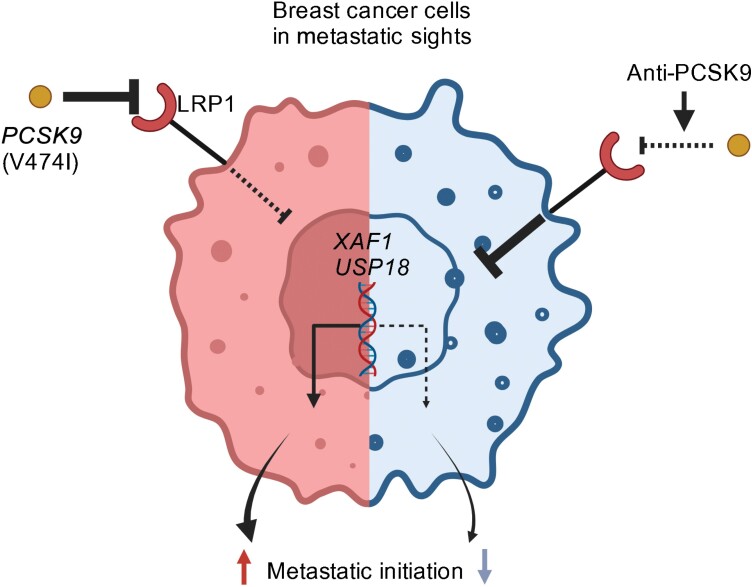
A human germline variant in *PCSK9* (V474I) causally drives breast cancer metastasis. Antibody-mediated therapeutic inhibition of PCSK9 suppresses breast cancer metastasis. The figure is created with Biorender.com.

The notion that germline genetic variations could shape metastatic outcomes represents a paradigm shift in cancer biology. Unlike somatic mutations, which arise during the evolution of the tumor, germline variants are inherited and present in every cell of the body [5]. This means that their effects are systemic, influencing not only the primary tumor but also the metastatic niche. Recent technological advances, including genome-wide association studies and next-generation sequencing, have begun to uncover germline variants associated with cancer risk and outcomes [6]. Among these, the proprotein convertase subtilisin/kexin type 9 (*PCSK9*) gene has garnered significant attention. PCSK9 is best known for its role in regulating cholesterol metabolism by promoting the degradation of low-density lipoprotein receptors [7]. However, emerging evidence suggests that PCSK9 may have broader biological functions, including the roles in immune modulation and cancer progression [8]. A specific missense variant of *PCSK9*, rs562556 (V474I), has recently been implicated in breast cancer metastasis, shedding light on the hereditary underpinnings of this lethal process [9]. In this study, Mei *et al*. identified the rs562556 missense germline variant in *PCSK9* as a key determinant of breast cancer metastatic outcomes [9]. This variant, characterized by a valine-to-isoleucine substitution at residue 474, was shown to be significantly associated with reduced survival in multiple breast cancer cohorts (Fig. 1). The V474I variant represents a gain-of-function mutation that enhances the activity of PCSK9, with profound implications for tumor biology. Approximately 70% of individuals of European ancestry are homozygous for this allele, underscoring its widespread prevalence and potential clinical relevance. Using a combination of clinical data, genetic modeling, and experimental approaches, the researchers demonstrated that the V474I variant promotes breast cancer metastasis through host-derived mechanisms. In large patient cohorts, homozygosity for the V474I allele was associated with a significantly higher risk of distant metastatic relapse compared to non-homozygous individuals. This association remained robust after adjusting for clinical confounders such as tumor stage, grade, and molecular subtype, suggesting that the variant’s effects are independent of traditional prognostic factors.

To establish causality, the researchers generated genetically engineered mouse models carrying the human *PCSK9* V474I variant. These mice exhibited a marked increase in metastatic colonization by breast cancer cells compared to their wild-type counterparts. Conversely, deletion of the *Pcsk9* gene in mice significantly suppressed metastasis in multiple experimental models, providing direct evidence that PCSK9 is a critical mediator of metastatic progression. Importantly, the pro-metastatic effects of PCSK9 were found to be independent of its canonical role in cholesterol metabolism, highlighting a novel and distinct function for this protein in cancer biology.

One of the most striking findings of the study was the identification of low-density lipoprotein receptor-related protein 1 (LRP1) as a key downstream target of PCSK9 in the metastatic process. LRP1 is a multifunctional receptor involved in diverse cellular processes, including endocytosis, signal transduction, and the regulation of extracellular matrix dynamics. In the context of cancer, LRP1 has been reported to influence cell migration, invasion, and survival [10]. However, its role in metastasis has remained poorly understood. Through a series of elegant experiments, the researchers demonstrated that PCSK9 promotes metastasis by downregulating LRP1 on the surface of breast cancer cells. Using mass spectrometry-based proteomics and flow cytometry, they showed that treatment with recombinant PCSK9 significantly reduced the abundance of LRP1 on the plasma membrane. This effect was more pronounced with the V474I variant, which exhibited enhanced binding to LRP1 compared to wild-type *PCSK9*.

By a CRISPR-guided targeting gene analysis, the authors revealed that the reduction in LRP1 levels was accompanied by the induction of a pro-metastatic gene signature, including interferon-stimulated genes (ISGs) such as *XAF1*, *USP18*, *ISG15*, and *OAS1A*. Functional assays provided compelling evidence for the role of these genes in metastatic progression. Depletion of *XAF1* and *USP18* in breast cancer cells significantly impaired their ability to form metastatic colonies *in vivo*, while overexpression of these genes enhanced metastasis. Notably, the expression levels of the *PCSK9*-*LRP1* gene signature correlated strongly with the rs562556 genotype in human breast cancer cohorts, suggesting that germline variation in *PCSK9* directly shapes the somatic gene expression landscape of tumors.

A previous study found that LRP1 suppresses inflammatory pathways by translocating the intracellular domain (ICD) into fibroblast nuclei. The present study further uncovered that LRP1 suppresses the expression of pro-metastatic genes by a similar mechanism. That is, the intracellular domain of LRP1 (LRP1-ICD) translocates to the nucleus, where it interacts with transcriptional regulators to repress the expression of ISGs in breast cancer cells. Loss of LRP1, either through PCSK9-mediated degradation or genetic deletion, abrogated this repression, leading to the activation of a pro-metastatic transcriptional program. This finding provides a mechanistic link between PCSK9 activity and the metastatic potential of breast cancer cells, offering new insights into the molecular underpinnings of metastasis.

Metastasis is a highly inefficient process, with the vast majority of disseminated tumor cells failing to establish secondary lesions. One of the major bottlenecks in this process is the ability of tumor cells to proliferate in distant organs under conditions of reduced attachment to the extracellular matrix [2]. The study revealed that PCSK9 enhances the proliferative competence of disseminated tumor cells, thereby facilitating their progression to macroscopic metastases. *In vitro* experiments using low-attachment culture conditions showed that recombinant PCSK9 significantly increased the proliferation of breast cancer cells. This effect was particularly pronounced with the V474I variant, which conferred a greater proliferative advantage compared to wild-type *PCSK9*. The researchers also observed a higher fraction of proliferating cells within metastatic colonies in mice expressing the V474I variant, consistent with its role in promoting metastatic initiation. Importantly, these effects were independent of PCSK9’s impact on cholesterol levels, as dietary cholesterol manipulation did not alter metastatic outcomes in mouse models.

Collectively, these findings highlight the multifaceted role of PCSK9 in metastatic breast cancer. By suppressing LRP1 and enhancing proliferative competence, PCSK9 orchestrates a complex program that enables tumor cells to overcome the barriers to metastasis. The identification of the V474I variant as a hypermorphic driver of these processes underscores the importance of germline genetics in shaping metastatic outcomes and provides a compelling rationale for targeting PCSK9 in high-risk breast cancer patients.

The discovery of PCSK9’s role in breast cancer metastasis opens new avenues for therapeutic intervention. PCSK9 inhibitors, originally developed for hypercholesterolemia, have shown promise in preclinical models of breast cancer. These findings suggest that PCSK9 inhibition could serve as a viable strategy to prevent or mitigate metastatic relapse in breast cancer patients. Importantly, the therapeutic effects of PCSK9 inhibition were independent of its cholesterol-lowering activity. This was evidenced by the lack of metastasis suppression with statin treatment, despite achieving similar reductions in cholesterol levels. This distinction underscores the specific role of PCSK9 in modulating tumor biology, which is separate from its function in lipid metabolism.

The identification of germline genetic influences in cancer progression dates back several decades. Early studies suggested that familial cancer syndromes, such as BRCA mutations in breast and ovarian cancers, were indicators of inherited cancer risks [11]. However, these syndromes predominantly focused on tumor initiation rather than metastatic spread. The current work on PCSK9 is groundbreaking because it shifts the paradigm, demonstrating that inherited factors can influence the latter stages of cancer progression. This revelation provides new insights into the natural history of cancer and highlights a previously underappreciated layer of complexity in cancer genetics. The intricate relationship between host genetics and tumor biology underscores the need to evaluate cancer as a systemic disease. The PCSK9-LRP1 axis exemplifies how host-derived factors can act as gatekeepers of metastatic progression. Unlike somatic mutations that arise within tumor cells, host genetic factors are omnipresent, influencing tumor behavior at multiple stages of the metastatic cascade. This systemic perspective opens the door for novel therapeutic strategies aimed at modulating host–tumor interactions, thereby disrupting the metastatic process at its roots.

The evolutionary implications of the *PCSK9* V474I variant extend beyond its immediate clinical relevance. The near fixation of this variant in certain populations suggests that it may have conferred a survival advantage in premodern environments. One hypothesis is that higher cholesterol levels associated with this variant provided an energy reserve that was beneficial during periods of food scarcity. However, in modern settings, where caloric abundance and sedentary lifestyles prevail, the same genetic traits may predispose individuals to chronic diseases such as cardiovascular disease and cancer. Understanding these evolutionary trade-offs can inform public health strategies and personalized medicine approaches.

The integration of germline genetic information into clinical practice represents a cornerstone of precision oncology. The *PCSK9* rs562556 variant serves as a prime example of how genetic markers can stratify patients based on their risk of metastatic relapse. By identifying individuals with high-risk genetic profiles, clinicians can tailor surveillance and treatment strategies to mitigate metastatic progression. Moreover, the availability of FDA-approved PCSK9 inhibitors offers a unique opportunity to translate these genetic insights into actionable interventions, bridging the gap between bench and bedside.

While the current findings are groundbreaking, they also raise several unanswered questions that warrant further investigation. For instance, the role of PCSK9 in other cancer types remains to be explored. Additionally, the potential synergistic effects of combining PCSK9 inhibitors with immune checkpoint therapies or targeted agents need to be systematically evaluated. Longitudinal studies tracking the impact of PCSK9 inhibition on patient outcomes over extended periods will be crucial for assessing the durability of therapeutic responses.

The discovery of the *PCSK9* rs562556 variant as a driver of breast cancer metastasis marks a significant milestone in cancer research. It challenges conventional wisdom by highlighting the role of germline genetics in shaping tumor progression and metastasis. By unraveling the complex interplay between host and tumor factors, this research paves the way for innovative therapeutic strategies and reinforces the importance of integrating genetic insights into clinical practice. As the field of oncology continues to evolve, the lessons learned from PCSK9 will undoubtedly inspire future breakthroughs in our quest to conquer metastatic disease.
